# Complementarity of ultrasound and fluorescence imaging in an orthotopic mouse model of pancreatic cancer

**DOI:** 10.1186/1471-2407-9-106

**Published:** 2009-04-08

**Authors:** Cynthia S Snyder, Sharmeela Kaushal, Yuko Kono, Hop S Tran Cao, Robert M Hoffman, Michael Bouvet

**Affiliations:** 1 Moores UCSD Cancer Center, University of California, La Jolla, CA, USA; 2 UCSD Department of Medicine, La Jolla, CA, USA; 3 UCSD Department of Surgery, La Jolla, CA, USA; 4 AntiCancer Inc., San Diego, CA, USA

## Abstract

**Background:**

Pancreatic cancer is a devastating disease characterized by dismal 5-year survival rates and limited treatment options. In an effort to provide useful models for preclinical evaluation of new experimental therapeutics, we and others have developed orthotopic mouse models of pancreatic cancer. The utility of these models for pre-clinical testing is dependent upon quantitative, noninvasive methods for monitoring *in vivo *tumor progression in real time. Toward this goal, we performed whole-body fluorescence imaging and ultrasound imaging to evaluate and to compare these noninvasive imaging modalities for assessing tumor burden and tumor progression in an orthotopic mouse model of pancreatic cancer.

**Methods:**

The human pancreatic cancer cell line XPA-1, engineered for stable, high-level expression of red fluorescent protein (RFP), was implanted into the pancreas of nude mice using orthotopic implantation. The tumors were allowed to grow over a period of one to several weeks during which time the mice were imaged using both fluorescence imaging and ultrasound imaging to measure tumor burden and to monitor tumor growth.

**Results:**

Whole-body fluorescence imaging and ultrasound imaging both allowed for the visualization and measurement of orthotopic pancreatic tumor implants *in vivo*. The imaging sessions were well-tolerated by the mice and yielded data which correlated well in the quantitative assessment of tumor burden. Whole-body fluorescence and two-dimensional ultrasound imaging showed a strong correlation for measurement of tumor size over a range of tumor sizes (R^2 ^= 0.6627, P = 0.003 for an exposure time of 67 msec and R^2 ^= 0.6553, P = 0.003 for an exposure time of 120 msec).

**Conclusion:**

Our findings suggest a complementary role for fluorescence imaging and ultrasound imaging in assessing tumor burden and tumor progression in orthotopic mouse models of human cancer.

## Background

Pancreatic cancer is a devastating disease with 5-year survival rates less than 5% [1]. In an effort to provide useful models for preclinical evaluation of drug therapy, we and others have developed orthotopic mouse models of pancreatic cancer [2-5]. These animal models promise to be invaluable for the testing of new cancer therapeutics. Orthotopically growing tumors can metastasize, in a pattern that resembles the clinical behavior of the original human tumor both in sites of metastasis and frequency of occurrence [6,7]. Tumor cells transduced and selected for high expression of fluorescent proteins implanted orthotopically can thus be used to visualize both primary and metastatic tumors [8]. Furthermore, the primary tumor and subsequent metastasis can be visualized non-invasively by whole body imaging through the skin of the nude mouse [3]. Such visualization can be a practical and convenient way to follow tumor growth and metastasis in real-time.

Other techniques of tumor imaging, including X-ray computed tomography (CT), positron emission tomography (PET), magnetic resonance imaging (MRI), and ultrasound, have been developed for small animal imaging and are available to investigators. Each technique has its specific advantages as well as limitations but, in the end, may be complementary to each other. For instance, we have previously shown that fluorescent protein imaging and MRI of pancreatic tumors are complementary and that there is a strong correlation between the two modalities [9]. Ultrasound imaging involves exposing tissues to high-frequency ultrasound waves (20–60 MHz in animals; 2–10 MHz in humans). It is a non-isotopic, noninvasive imaging modality which provides good soft tissue contrast and yields a high degree of spatial resolution without a requirement for contrast agents. This noninvasive technique produces a dynamic real-time image of the tissue from which structural and functional information can be obtained. Ultrasound in mice has been used to monitor tumor growth in prostate cancer [10,11], mouse mammary tumors [12], and ovarian cancer [13]. In this study, we sought to determine if fluorescence imaging and ultrasound imaging of orthotopic pancreatic tumors would correlate and potentially be a useful combined modality for monitoring of tumor growth, off-setting some of the limitations of each modality used alone.

## Methods

### Cell Culture

The human pancreatic cancer cell line XPA1 was a gift from Dr. Anirban Maitra at Johns Hopkins University. Cells were maintained in RPMI 1640 media supplemented with 10% fetal bovine serum (FBS) and 2 mM glutamine from (Gibco-BRL, Life Technologies, Inc., Grand Island, NY). All media was supplemented with penicillin/streptomycin (Gibco-BRL), L-glutamine (Gibco-BRL), MEM nonessential amino acids (Gibco-BRL), sodium bicarbonate (Cellgro, Herndon VA), and sodium pyruvate (Gibco-BRL). All cell lines were cultured at 37°C with 5% CO_2_.

### RFP Retroviral Transduction and Selection

The pDSRed-2 vector (Clontech Laboratories, Inc., Palo Alto, CA) was used for stable expression of red fluorescent protein (RFP) in the human pancreatic cancer cell line XPA-1. The pDsRed-2 retrovirus, which also contains a neomycin resistance gene, was produced in PT67 packaging cells. 20% confluent XPA-1 cells were incubated with retroviral supernatants of the packaging cells for 24 hours. Fresh medium was then replenished and the cells were allowed to grow for another 12 hours. This was repeated until high levels of RFP expression were observed under fluorescence microscopy. Cells were then trypsinized and harvested, and subcultured in selective media containing Geneticin G418 (Invitrogen Corp., Carlsbad, CA). The level of G418 was increased in a stepwise fashion from 200 μg/mL to 2000 μg/mL. Clones with high RFP expression were isolated using cloning cylinders and grown for 10 passages in the absence of G418 to select for stable *in vitro *expression of RFP.

### Animal Care

Athymic *nu/nu *nude mice between 4 and 6 weeks of age were maintained in a barrier facility on high efficiency particulate air (HEPA)-filtered racks. The animals were fed with autoclaved laboratory rodent diet (Teckland LM-485; Western Research Products, Orange, CA). All animal studies were approved by the UCSD Institutional Animal Care and Use Committee and conducted in accordance with the principles and procedures outlined in the NIH Guide for the Care and Use of Animals.

### Orthotopic Tumor Implantation

Orthotopic human pancreatic cancer xenografts from the pancreatic cancer cell line XPA-1-RFP were established in nude mice by orthotopic implantation. Orthotopic implantation was performed with the animals anesthetized by intramuscular injection of 0.02 mL of a solution of 50% ketamine, 38% xylazine and 12% acepromazine maleate. The animals were anesthetized and a small transverse incision was made in the left lateral flank through the skin and peritoneum. The tail of the pancreas was exposed and 5 × 10^5 ^(20 mice) or 2 × 10^6 ^(15 mice) XPA-1-RFP cells were injected into the pancreas in a total volume of 20 μL of serum-free media and Matrigel (1:1) using a Hamilton syringe (Sigma-Aldrich). The pancreas was then returned to the abdomen and the peritoneum and skin were closed using 6-0 Polysorb surgical suture (US Surgical Corporation, Norwalk, CT).

### Fluorescence Imaging

Mice were imaged 1–2 times per week using the Olympus OV100 Small Animal Imaging System (Olympus Corp, Tokyo Japan) containing an MT-20 light source (Olympus Biosystems, Planegg, Germany) and either the DP71 CCD camera (Olympus Corp. Tokyo, Japan) for qualitative color images of tumor implants or with the Hamamatsu monochrome camera (Hamamatsu Corp, Hamamatsu City Japan) for quantitative evaluation of fluorescence intensity. For whole body fluorescence imaging of live mice, the mice were anesthetized by inhalation of 2–3% isoflurane with 1% oxygen during the imaging session. Mice were imaged with their ventral sides towards the camera, such that images used to determine tumor sizes were all obtained in the coronal plane. Fluorescence images were collected at a predetermined series of exposure times using RFP filter sets with an excitation filter of 545/30 and an emission filter of 598/55. All images were processed for contrast and brightness and analyzed using ImageJ http://rsb.info.nih.gov/ij/download.html software and Photoshop Elements-4 (Adobe Systems Inc, San Jose, CA) software. Tumor margins were determined by ImageJ software from monochrome fluorescence images using automated thresholds based on pixel intensity. The OV100 is calibrated by the manufacturer to display a scale bar on acquired images. This scale bar is then used to measure lengths and calculate cross-sectional areas using either OV100 software or ImageJ software.

### Ultrasound Imaging

Ultrasound imaging was performed on the same day as fluorescence imaging. Mice were anesthetized by inhalation of 2–3% isoflurane with 1% oxygen. Anesthetized mice were placed on a thermostatically controlled heating pad to maintain body temperature. Tumors were imaged with the VisualSonics Vevo™ 770 *In Vivo *High-Resolution Micro-Imaging System (VisualSonics Inc, Toronto, Ontario, Canada). With mice lying on their right sides, an aqueous ultrasonic gel was applied to the skin overlying the spleen and subjacent pancreas. A transducer with central frequency at 40 MHz, providing axial resolution of 40 μm with a 14.6 mm field of view, was used for imaging of smaller tumors. A transducer with central frequency at 25 MHz transducer, providing axial resolution of 70 μm with a 20 mm field of view, was used for larger tumor imaging. Cine loops of ultrasound image were recorded digitally and reviewed. An image frame showing the tumor at it's largest cross-sectional size was selected for analysis. The largest tumor area in a coronal plane was measured by manually tracing the margin of the tumor using Vevo 770 software. The software then calculates the area enclosed within the delineated region. The instrument is calibrated to allow measurements to be determined accurately.

### Correlating fluorescence imaging and ultrasound imaging measurements of tumor size

To assess the correlation of fluorescence and ultrasound imaging modalities for assessment of pancreatic tumor size and tumor growth, all orthotopically implanted pancreatic tumors, from a total of 29 mice, were imaged using both imaging modalities. Each tumor was imaged *in vivo *to determine its size (maximum cross sectional area) in the coronal plane, as described above. Mice were sacrificed at predetermined times or when the dimensions of their tumors reached various target size ranges. A subset of mice were imaged on a weekly basis for four weeks so that the growth of individual tumors could be followed. For each imaging modality, raw imaging data was saved at the end of each imaging session and batch processed at a later time, blinding the data analysis process to individual tumor identity. Tumor size data calculated from fluorescence and ultrasound images was then compiled in a spreadsheet for comparison and analysis. To determine the correlation between imaging modalities, we compared tumor size calculated from whole-body fluorescence images acquired using a single exposure time to tumor size determined from ultrasound images. This process yielded multiple correlation graphs, one for each exposure time. The correlation coefficient R^2 ^was calculated between ultrasound tumor area and whole-body fluorescence tumor area. P < 0.05 was considered significant. Statistics were performed using Excel software (Microsoft Corporation, Redmond, WA).

### Gross and histologic examination of tissues

At necropsy, larger tumors were dissected away from the normal pancreas and measured *ex vivo *using calipers to determine three-dimensional size. A subset of small tumors, those imageable by fluorescence and/or ultrasound but not apparent to the naked eye when looking at the intact pancreas, were subjected to histologic examination for confirmation of imaging findings. Additional tumors were processed for histological examination as necessary to address specific anatomic and micro-anatomic questions. Fresh tissues were placed in Bouin's fixative and processed using standard methods for paraffin embedding and sectioning. Sections were stained used hematoxylin and eosin and examined using a inverted Nikon DE-300 fluorescence microscope equipped with a Spot RD camera (Diagnostic Instruments, Inc., Sterling Heights, MI).

## Results and Discussion

### Imaging systems

**Figure 1:**
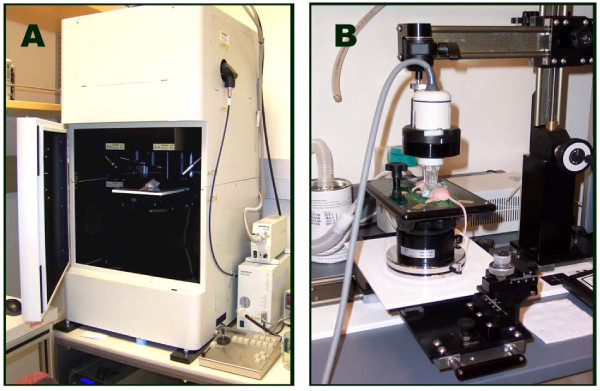
**Imaging hardware used in this comparison study**. **A**. Olympus OV100 Small Animal Imaging System. **B**. VisualSonics Vevo™ 770 *In Vivo *High-Resolution Micro-Imaging System.

Figure 1 shows the imaging systems used in this study. The Olympus OV100 Small Animal Imaging System (Figure 1A) contains an internal platform on which the anesthetized mice are placed during imaging. The camera is positioned above the mice, and images are obtained in the same plane as the platform. A diagram of the Olympus OV100 optical layout and additional details regarding the OV100 have been previously published [14]. The VisualSonics Vevo™ 770 *In Vivo *High-Resolution Micro-Imaging System (Figure 1B) also has a platform on which anesthetized mice are placed during imaging. The ultrasound imaging probe yields images which are perpendicular to the plane of the platform. We generated coronal images for both fluorescence and ultrasound imaging modalities for comparison of tumor size. Additional information concerning the Vevo™ 770 is available from the VisualSonics website http://www.visualsonics.com/products/products_vevo770.htm.

### Measurement of tumor size using fluorescence imaging and ultrasound imaging

**Figure 2:**
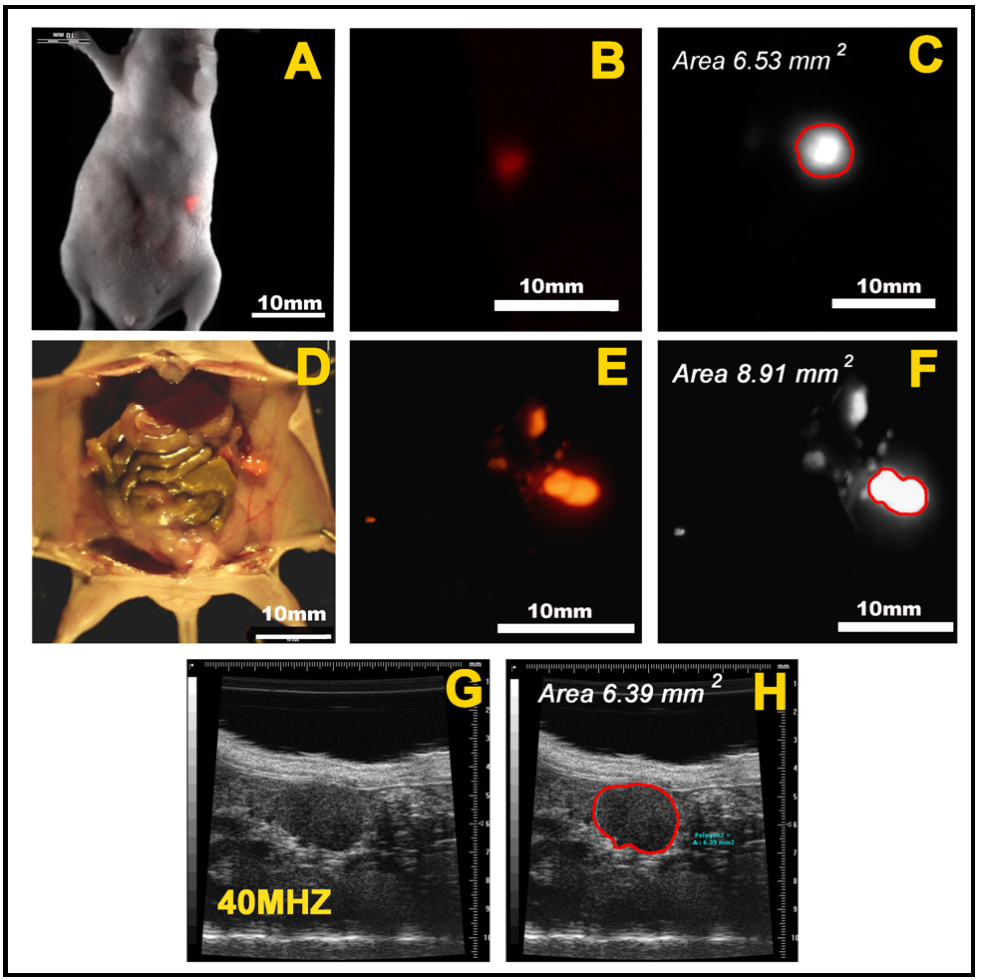
**Fluorescence and ultrasound imaging of orthotopic pancreatic tumor implants and measurement of tumor size**. **A-H**. This mouse was imaged two weeks after orthotopic injection of a human pancreatic cell line (XPA-1) stably expressing red fluorescent protein (RFP). **A-C**. Evaluation of tumor size by whole body imaging of an anesthetized mouse. **A**. Bright field and color fluorescence images were overlaid to show the coronal image of the fluorescent tumor. **B**. Enlarged whole body fluorescence image of tumor. This image was obtained using a color (non-quantitative) fluorescence imaging camera. **C**. Monochrome (quantitative) fluorescence image of the tumor shown in panels A and B. This coronal image was analyzed using ImageJ software to determine the cross-sectional size of the tumor in millimeters squared (mm^2^). **D-F**. Evaluation of tumor size at necropsy. **D**. Overlay of bright field and color fluorescence images showing the fluorescent tumor implant at necropsy. **E**. Enlarged color fluorescence-only image of the tumor as shown in D. **F**. As for panel C above, the cross-sectional size of the tumor shown in panels D and E was determined using ImageJ software. **G**. This ultrasound image of the pancreatic tumor mass was obtained using a Visual Sonics Vevo 770 small animal ultrasound imaging system. With the anesthetized mouse in a lateral position, the 40 MHz ultrasound transducer was oriented to obtain coronal image slices of the tumor. **H**. Image showing the tumor at its maximum cross sectional size in the coronal plane. Vevo 770 imaging software was used to determine the cross sectional size (in mm^2^) of the tumor.

Whole-body fluorescence imaging and ultrasound imaging both allowed for the visualization and measurement of orthotopic pancreatic tumor implants *in vivo*. Figure 2 shows the process used to evaluate and measure tumors. A color camera was used to collect bright-field images of the mice and color images of the RFP-expressing orthotopic tumor implants, which were imaged through the intact skin (Figure 2B). Bright-field and fluorescence images were overlaid to generate a composite image showing the position of the fluorescent tumor relative to the mouse (Figure 2A). For measurement of tumor size, the color camera was replaced with a monochrome camera capable of quantitative fluorescence imaging. Monochrome images were analyzed using ImageJ software to calculate the cross-sectional size in mm^2^, as shown in Figure 2C.

Figure 2G shows an ultrasound image slice of a small orthotopic tumor implant. Vevo 770 software was used to manually trace the borders of the tumor on the image and to calculate tumor size in mm^2^, as shown in Figure 2H.

Following ultrasound and whole-body fluorescence imaging, the abdominal cavities of sacrificed mice were opened and examined, the pancreas was exposed, and the opened abdominal cavity was subjected to fluorescence imaging. Fluorescence-only images (Figure 2E), composite bright-field and color fluorescence images (Figure 2D), and monochrome (quantitative) fluorescence images (Figure 2F) were generated. For small tumors, the size of the tumor was calculated as described above from open or *ex vivo *fluorescence images, using ImageJ software to determine tumor size in mm^2^. There is a very close correlation between whole-body fluorescence and ultrasound imaging for measurement of tumor size *in vivo *using this approach. The somewhat larger tumor size determined at necropsy for this tumor most likely reflects oblique imaging of this elongate tumor.

### Comparison of whole-body fluorescence imaging with ultrasound imaging for monitoring tumor growth over time

**Figure 3:**
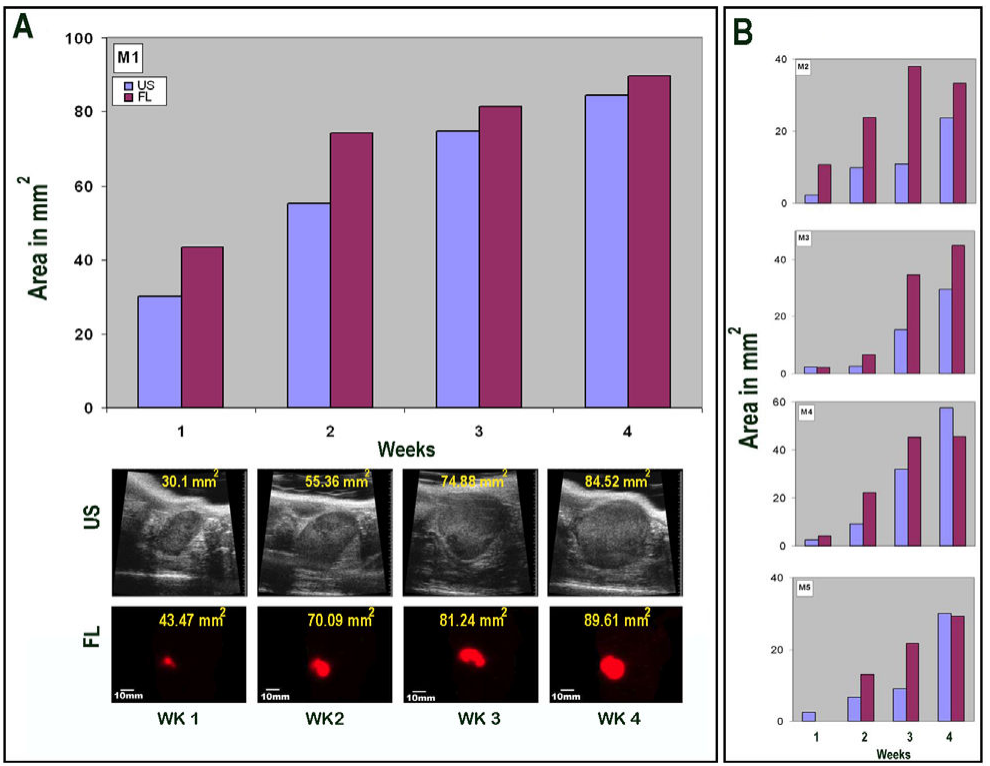
**Comparison of whole body fluorescence imaging and ultrasound imaging to monitor tumor growth over time in individual mice**. Whole-body fluorescence and ultrasound images were obtained on a weekly basis for a period of four weeks. Tumor measurements were determined as explained in the legend for Figure 2. **A**. The graph shows the growth of an orthotopic pancreatic-tumor implant in a single mouse (M1) as assessed by both whole-body fluorescence (FL) and ultrasound (US) imaging. The four corresponding ultrasound (upper row) and color fluorescence (lower row) images are shown below the graph with the calculated tumor sizes (mm^2^) for each image indicated. **B**. Graphical depictions of tumor growth over a four week period for an additional four mice (M2 thru M5) with orthotopic pancreatic tumor implants. Bar graphs shown in this figure depict single measurements taken at weekly timepoints. The graphs demonstrate a strong correlation between whole-body fluorescence and ultrasound imaging modalities for determination of tumor size.

Figure 3A shows growth of an orthotopic pancreatic tumor implant over a 4-week period following orthotopic implantation of XPA-1-RFP human pancreatic tumor cells. The graph demonstrates a high correlation between whole-body fluorescence imaging and ultrasound imaging for monitoring tumor growth over time. The four images obtained using each imaging modality demonstrate that the growing pancreatic tumor implants are clearly visualized using both fluorescence and ultrasound imaging. Figure 3B shows the data obtained from four additional mice serially-imaged to monitor growth of orthotopic pancreatic tumor implants. There was a high degree of variability in the absolute size and the growth kinetics of individual tumors between mice originally injected with the same number of XPA-1-RFP tumor cells (Figure 3B), independent of imaging modality.

### Comparison of whole-body fluorescence imaging with ultrasound imaging for determination of tumor size over a range of tumor sizes

**Figure 4:**
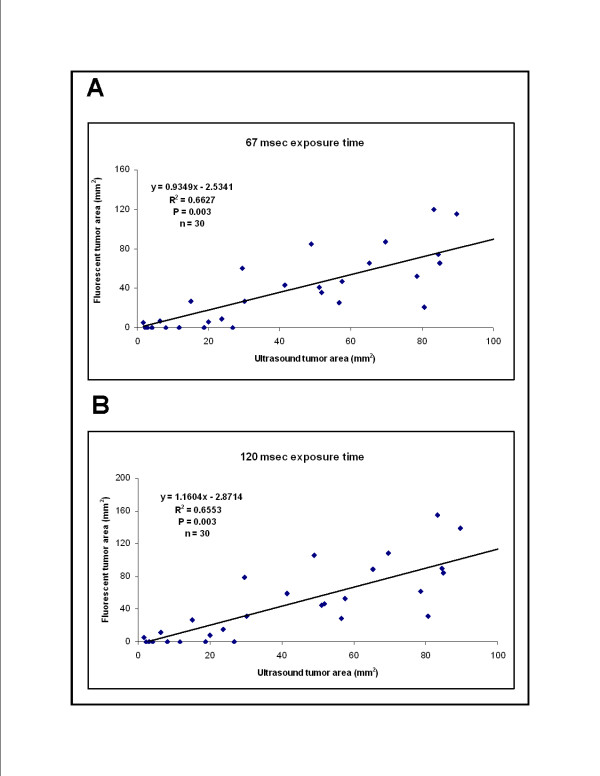
**Correlation of whole body fluorescence imaging and ultrasound imaging for assessment of tumor size in mice with orthotopic implants of fluorescently-labeled pancreatic tumor cells**. Mice harboring tumors of varying sizes (n = 30) were imaged using both fluorescence and ultrasound imaging to assess tumor size. The resulting measurements (cross-sectional area in mm^2^, determined as previously explained) for each tumor were graphed to determine how well the imaging modalities correlated for assessment of tumor size over a range of tumor sizes. We assessed tumor sizes at two exposure times, 67 msec and 120 msec, that gave clear visual images which were neither under-exposed nor over-saturated and analyzed these images to measure tumor size. **A-B**. Determination of tumor size from fluorescence images taken with either a 67-millisecond exposure time (panel A) or a 120-millisecond exposure time (panel B). There is a strong correlation between fluorescence and ultrasound imaging modalities for assessment of tumor size over a range of tumor sizes at both exposure times. Both exposure times (67 msec and 120 msec) yielded similar values for R^2 ^and P.

As expected, fluorescence images obtained using higher exposure times yielded larger calculated tumor sizes. Exposure times of 67 msec and 120 msec yielded correlation slopes (m = 0.9349 and m = 1.1604, respectively) which came closest to showing a perfect correlation between imaging modalities for assessment of tumor size. This data is shown in Figure 4. Whole-body fluorescence and ultrasound imaging showed a strong correlation for measurement of tumor size over a range of tumor sizes (R^2 ^= 0.6627, P = 0.003 for an exposure time of 67 msec and R^2 ^= 0.6553, P = 0.003 for an exposure time of 120 msec). Data were obtained from tumors of various sizes up to a limiting size of about 150 mm^2^. Tumors > 150 mm^2 ^were too large to fit within a single ultrasound image frame and would, therefore, have been difficult to measure by ultrasound imaging in this study. Tumor dimensions were determined at necropsy on 22 tumors. Tumor volumes, which were approximated using the formula for an ellipsoid, were compared to tumor sizes determined by imaging studies. *In vivo *imaging studies showed a strong correlation with post mortem analysis for determination of tumor size (R^2 ^= 0.7782 and R^2 ^= 0.6092 for ultrasound imaging and fluorescence imaging, respectively; P < 0.001 for both).

### Correlation of *in vivo *ultrasound imaging and *ex vivo *fluorescence imaging with final histopathology

**Figure 5:**
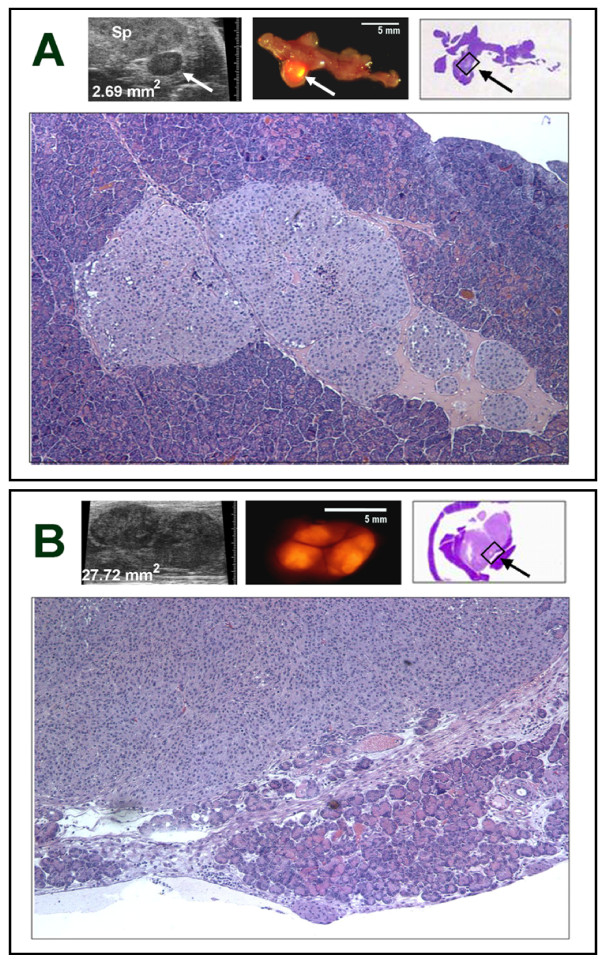
**Correlation of histopathology with *ex vivo *fluorescence and *in vivo *ultrasound images in mice with orthotopic implants of fluorescently-labeled pancreatic tumor cells**. **A**. Ultrasound image of a small orthotopic tumor implant (upper left image; tumor indicated by arrow; Sp, spleen) whose presence was confirmed by *ex vivo *fluorescence imaging (upper middle image). The histopathologic findings correlated well with the ultrasound and *ex vivo *fluorescence findings, as shown here in this low power (1× magnification) hematoxylin- and eosin-stained permanent tissue section (upper right image). A higher magnification (100×) of the area bordered by the black rectangle in the low power image is also shown (lower image). **B**. Ultrasound imaging (upper left image) documented the bilobate tumor shown here, *Ex vivo *fluorescence imaging also demonstrated the bilobate tumor (upper middle image) contained within the pancreas. Histologic examination showed close juxtapositioning of dual tumor masses surrounded by a stretched capsule of normal pancreas (upper right image). This low power (1× magnification) image also shows the adjacent spleen, which was removed with the pancreas as an organ block to aid in orientation of the fixed tissue for histologic examination. A higher magnification (100×) of the area bordered by the black rectangle in the low power image is shown enlarged below to better demonstrate the tumor mass and the normal pancreas tissue surrounding it.

Findings obtained from ultrasound imaging of live mice and *ex vivo *fluorescence imaging of tumor implants at necropsy were correlated with histopathologic findings, as shown in Figure 5. The presence of a small (2.69 mm^2^) tumor implant detected by ultrasound imaging of this mouse (Figure 5A) was confirmed by *ex vivo *fluorescence imaging of the pancreas. The histopathologic findings for this tumor correlate perfectly with the ultrasound and *ex vivo *fluorescence findings. A somewhat larger tumor (total area 27.72 mm^2^) appeared to be bilobate in ultrasound images (Figure 5B). The bilobate form of the tumor was confirmed by *ex vivo *fluorescence imaging and well demonstrated by histopathologic studies.

### Strengths and weaknesses of whole-body fluorescence imaging and ultrasound imaging for evaluation of orthotopic pancreatic tumor implants

In this study, ultrasound imaging showed a modest advantage over whole-body fluorescence imaging for detection of small pancreatic tumor implants (tumors < 25 mm^2^, Figure 4). Ultrasound imaging of small implants was facilitated by knowledge of their expected location subjacent the spleen; ultrasound scanning was focused on this area in search of tumor. In contrast, the spleen concealed small orthotopic tumor implants lying directly beneath it from whole-body fluorescence imaging. The presence of these small tumors was verified by open or *ex vivo *fluorescence imaging (Figure 2F). In this setting, the high specificity of fluorescence imaging for RFP-expressing tumor cells proved an advantage, allowing detection of small satellite tumor foci that were easily overlooked using ultrasound imaging. Larger tumors, particularly those with multiple lobes or convoluted surfaces, would be difficult to measure by two-dimensional ultrasound imaging and, therefore, were excluded from analysis. These larger tumors were easily imaged by whole-body fluorescence imaging.

## Conclusion

*In vivo *imaging studies yield information that cannot be obtained from postmortem analysis alone, such as tumor growth rates, the time course of metastases, or tumor response to experimental therapeutics. Whole-body fluorescence imaging and ultrasound imaging are both noninvasive imaging modalities with good sensitivity for detection of orthotopic pancreatic tumor implants in nude mice. The mice in this study tolerated repeated imaging sessions well, which likely reflects the use of an inhalation anesthetic rather that an injectable one and the noninvasiveness of the imaging modalities. The experimental findings presented herein are specific to the XPA-1-RFP orthotopic pancreas tumor model, but our observations have general applicability. Both imaging modalities should prove effective for preclinical studies of cancer progression in other orthotopic mouse models of cancer.

A number of factors should be considered when deciding to use either fluorescence or ultrasound imaging methods to monitor tumor growth in longitudinal studies of experimental mouse models. Both imaging modalities require that the skin overlying the imaged area be hairless. When using hairy mice rather than nude mice, the hair should be removed using a depilatory lotion prior to imaging. Detection sensitivity falls off for both imaging modalities when tumors are very small or lie deep within the abdomen. This limitation is particularly relevant to studies in which the goal is to follow early stages of metastasis through whole body imaging. In the orthotopic model of human pancreatic cancer used this study, we implanted fluorescent XPA-1-RFP cells, which stably express high levels of RFP. The bright expression of RFP, combined with its favorable emission spectrum, facilitated detection of tumors in whole-body fluorescence imaging studies. Sensitivity for detection of fluorescent tumors will be diminished in cell lines showing less robust or less stable expression of fluorescence and when using fluorophores other than RFP for which autofluorescence by non-expressing, normal host tissues is increased. On the other hand, fluorophores with longer wavelength excitation and/or emission spectra would be expected to have higher tissue penetration than RFP. However, fluorescence intensity and cellular toxicity are also variables which effect the utility of a fluorophore for *in vivo *experimentation and must also be evaluated.

In this study, orthotopic tumors arising from XPA-1-RFP implants grew with well-demarcated borders yielding sharp outlines on ultrasound images. This factor was helpful for identification and measurement of the tumors, particularly the smaller tumors. Tumors which grow with infiltrating margins will be more difficult to identify and to measure in ultrasound studies. As it is for fluorescence imaging, tissue penetration is also an issue for ultrasound imaging. Ultrasound transducers which produce longer wavelength sound waves yield greater tissue penetration but at the expense of imaging resolution. A variety of transducers are available to optimize this balance. Different transducers are used for small animal imaging as for imaging of patients in the clinic. In both settings, tissue penetration and imaging resolution counterbalance each other. A variety of contrast agents are under investigation for use in ultrasound imaging. These agents will likely prove helpful in certain settings.

In short, the relative merits of each fluorescence and ultrasound imaging modalities will be influenced by characteristics unique to each tumor-host model system. However, our findings suggest that fluorescence imaging and ultrasound imaging modalities are complementary approaches for monitoring tumor progression and treatment response in preclinical studies using orthotopic mouse models of human cancer.

## Competing interests

The authors declare that they have no competing interests.

## Authors' contributions

CSS, SK, and MB designed the experiments and analyzed the data. CSS and SK performed the experiments. SK was responsible for data management and preparation of figures. YK and HSTC helped with performance of experiments, with data analysis, and with editing of the manuscript. CSS and MB wrote the manuscript. RMH provided valuable experimental resources and helped with data analysis and with editing of the manuscript.

## Pre-publication history

The pre-publication history for this paper can be accessed here:

http://www.biomedcentral.com/1471-2407/9/106/prepub
